# Widespread subcutaneous necrosis in spotted fever group Rickettsioses from the coastal belt of Sri Lanka- a case report

**DOI:** 10.1186/s12879-017-2375-z

**Published:** 2017-04-17

**Authors:** Nathasha Luke, Hasini Munasinghe, Lakshmi Balasooriya, Ranjan Premaratna

**Affiliations:** 1 Department of Pharmacology, Faculty of Medicine, University of Kelaniya, Kelaniya, Sri Lanka; 2 Professorial Medical Unit, Colombo North Teaching Hospital, Ragama, Sri Lanka; 3 Department of Medicine, Faculty of Medicine, Ragama, Sri Lanka

**Keywords:** Spotted fever group rickettsioses, Skin necrosis, Purpura fulminans, Fern leaf rash, Diagnosis, Case report

## Abstract

**Background:**

Spotted fever group rickettsioses (SFGR) transmitted mostly by ticks are increasingly discovered around the World and some of them are either re-emerging or emerging in Sri Lanka. Accidental human infections caused by these vector borne zoonotic diseases generally give rise to nonspecific acute febrile illnesses which can be complicated by multi organ involvement carrying high morbidity and mortality. Nonspecific clinical features and non-availability of early diagnostic facilities are known to result in delay in the diagnosis of rickettsial infections. Therefore, awareness of their prevalence and more importantly their clinical features would be help in the early diagnosis and institution of appropriate therapy.

**Case presentation:**

A 39-year-old otherwise healthy female presented with an acute febrile illness complicated by severe small joint and large joint arthritis, jaundice, acute kidney injury and disseminated intravascular coagulation (DIC) mimicking palindromic rheumatism or severe sepsis. She later developed a widespread fern-leaf pattern necrotic skin rash with evidence of vasculitis on the palms and soles, aiding the clinical diagnosis of SFGR. She had very high antibody titres against *R. conorii* antigen confirming the diagnosis and recovered completely with anti-rickettsial therapy.

**Conclusion:**

We feel that clinicians should be aware of the unusual clinical presentations such as purpura fulminans and ‘fern-leaf’ pattern necrotic skin rash of SFGR infection. Such knowledge would not only benefit those who practice in tropics with limited diagnostic facilities but also would improve the management of acute febrile illness in returning travelers who visit endemic areas.

## Background

Spotted fever group rickettsioses (SFGR) have a wide global distribution and consist of more than 30 species or subspecies [1]. Since the use of molecular tools for detection, human pathogenic species of SFGR are increasingly described [1]. While most of these organisms are transmitted by ticks and in some cases by mites that generally feed on wild or domestic animals, the infection in human is accidental [2]. Infections result in an acute febrile illness with undifferentiated clinical features. However, presence of a vasculitic rash that involves the body and the extremities during the course of illness together with a history of tick bites or exposure risk contributes to a clinical diagnosis [3]. Patients can have various organ involvement such as pneumonitis, hepatitis, acute renal failure, encephalitis and when severe, may lead to multiple organ involvement carrying high mortality [4].

In Sri Lanka, rickettsial infections have been increasingly reported over the last 2-3 decades, and they include scrub typhus (ST) caused by *Orientia tsutsugamushi* and spotted fever group rickettsioses [5–7]. Although SFGR was described less commonly in the Western province of Sri Lanka, in a hospital based study where patient recruitment had been on strict inclusion criteria [5], with the establishment of Indirect Immunofluorescence antibody (IFA) based diagnostics it became apparent that SFGR are as common as scrub typhus (unpublished data). Furthermore, most cases of SFGR present sporadically throughout the year with no seasonal pattern compared to more seasonal outbreaks of ST (unpublished personal observation). Although the organisms causing SFGR in the Western Province of Sri Lanka are yet to be identified, dog ticks are suspected as the most likely vector transmitting the illness based on the reported experiences of patients.

Nonspecific clinical features and non-availability of early diagnostic facilities have resulted in delay in the diagnosis of rickettsial infections. Therefore, awareness of their prevalence and more importantly their clinical features would be help in the early diagnosis and institution of appropriate therapy.

In SFG rickettsioses, subcutaneous necrosis and digital gangrene most likely related to small-vessel occlusion have been described in severe late stage infections caused by *Rickettsia rickettsii* (Rocky Mountain spotted fever) and *Rickettsia conorii* [8–11]. Such severe form of skin involvement is described as Purpura fulminans (PF) and include a heterogeneous group of disorders characterized by rapidly progressive purpuric lesions that may develop into extensive areas of skin necrosis, and peripheral gangrene. This rare disorder is associated with laboratory evidence of consumptive coagulopathy and is often fatal [11]. PF is usually associated with many infections, most notably with meningococcal, staphylococcal, streptococcal infections in addition to SFG rickettsioses [11]. Probable less severe or early form of PF; termed as “fern leaf pattern” skin rash has been previously reported in elderly patients with SFG rickettsiosis in the central hills of Sri Lanka [3, 6, 7, 12].

We present a sporadic case of SFGR infection from coastal belt of Sri Lanka who presented with an acute febrile illness and polyarthritis complicated with acute kidney injury and DIC. She probably developed early PF in the form of wide spread ‘fern-leaf’ pattern necrotic skin rash with evidence of vasculitis on the palms and soles and DIC on the 10th day of illness. We feel that clinicians should be aware of this unusual clinical presentation that could occur in association with SFGR infection. Such knowledge would not only benefit those who practice in tropics with limited diagnostic facilities but also would improve the management of acute febrile illness in returning travelers who visit endemic areas.

## Case presentation

A 39-year-old housewife, a mother of two children, presented with a history of high intermittent fever (101-103 °F), chills and rigors for eight days. Apart from mild intermittent asthma, she had no other co-morbidities. The intensity and frequency of fever had increased despite treatment with oral co-amoxyclav by a general practitioner. She had associated severe frontal headache, nausea and vomiting. However, the most striking feature was that she developed progressively severe pain in almost all large and small joints of the body with backache and neck pain limiting her movements. As a result, at the time of presentation she was bedbound. In addition, she had noted yellowish discoloration of the sclerae and dark urine around the third day of fever. She did not have any similar clinical manifestations in the past or any clinical signs suggestive of a connective tissue disease. She had an intrauterine contraceptive device for one year. Although menstrual periods were regular, she complained of intermittent offensive vaginal discharge for the past few months. She denied history of recent pregnancy or gynecological interventions.

She had been a victim of floods two weeks prior to the onset of current illness. However, she had not travelled away from her residence. She had no contact with farm animals but had two pet dogs at home.

On examination she looked very ill, icteric, pale and dehydrated. She had severe neck stiffness and bilateral symmetrical large and small joint arthritis with distal interphalangeal joint sparing. Careful physical examination on admission did not reveal skin rash, oral ulcers or lymphadenopathy. Abdominal examination was unremarkable except for a non-tender enlarged liver 2 cm below the right costal margin. She had tachycardia of 100 beats/min and blood pressure of 110/60 mmHg with no cardiac murmurs. She was not tachypneic, and her lungs were clear. Vaginal examination was unremarkable.

The differential diagnosis included leptospirosis, a palindromic rheumatism with systemic involvement or severe bacterial sepsis with a meningitic process. Admission investigations revealed a WBC count of 12.7 × 10^3^/mL (Neutrophils 80% Lymphocytes 16%) and c-reactive protein (CRP) of 327 U/L (Normal < 5 U/L). Blood picture showed toxic granules in polymorphonuclear leukocytes together with a left shift suggesting a bacterial infection. Urinalysis on admission showed 2+ proteinuria with occasional leukocytes and red blood cells. Serum creatinine was 229 μmol/L (normal 45-90 micromol/L), aspartate transaminase (AST) 92 U/L(normal 0 – 35 U/L), alanine transaminase (ALT) 45 U/L (normal 0 – 35 U/L), bilirubin 95 μmol/L (normal 5.0–17.0 μmol/L), Direct 40 μmol/l. Serum alkaline phosphatase was 255 U/L(normal 50-100 U/L), her ESR was 72 mm/1^st^hr, and anti-nuclear antibodies and rheumatoid factor were negative. Urine culture was negative, and the blood culture were negative.

While awaiting investigation results, she was treated as for leptospirosis or for severe sepsis with intravenous ceftriaxone, 1 g twice daily together with intravenous hydration, antipyretics and analgesics.

**Fig. 1:**
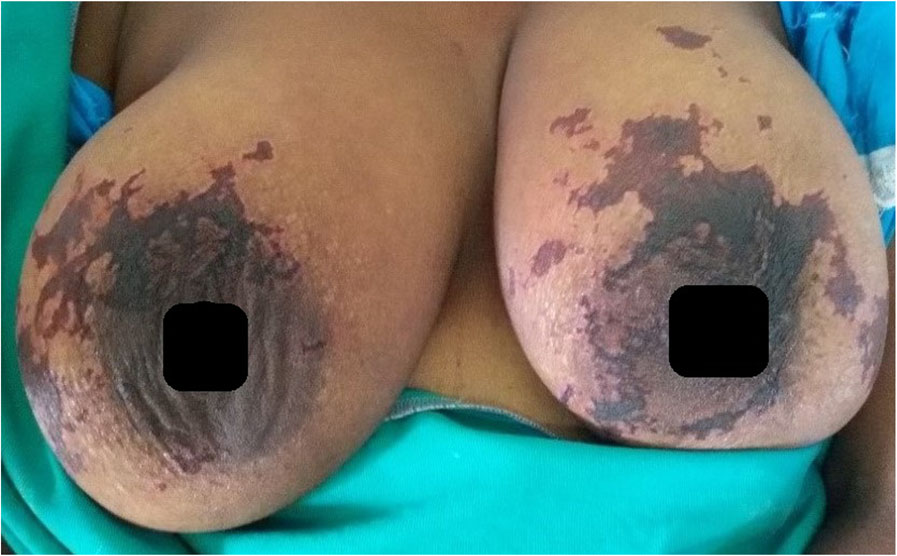
Patches of subcutaneous necrosis over the breasts

**Fig. 2:**
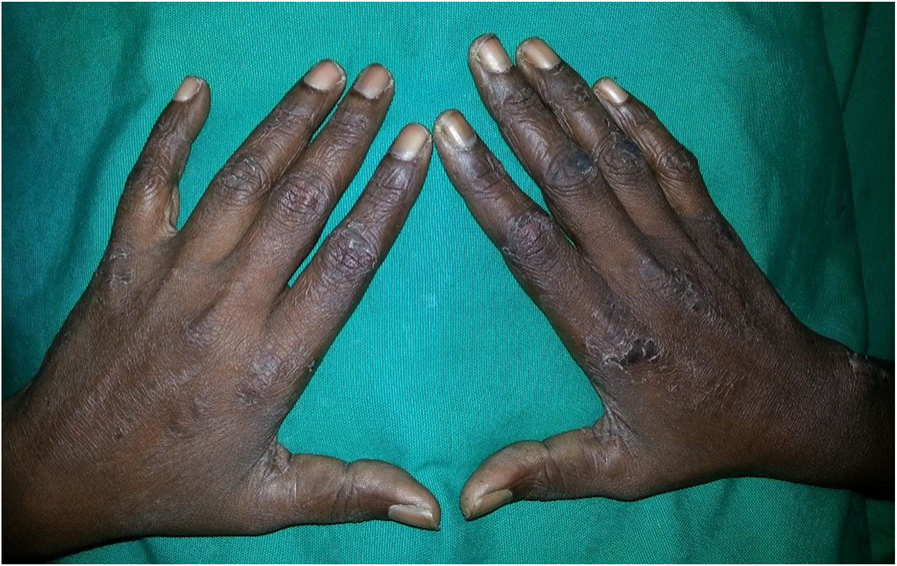
Subcutaneous necrosis mainly involving the dorsal aspects of the joints of hands

**Fig. 3:**
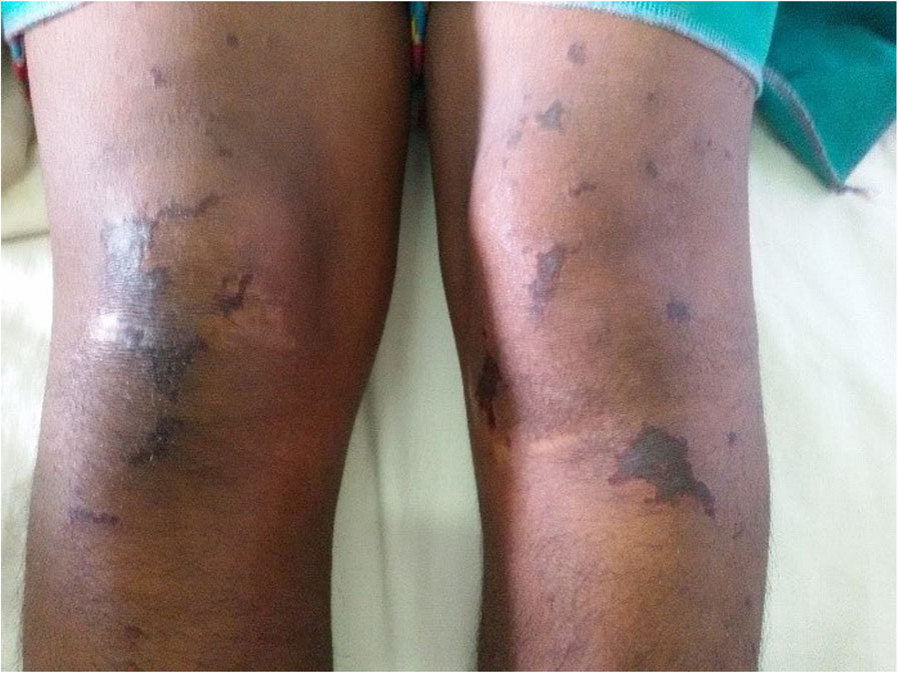
Areas of subcutaneous necrosis over the thighs and the knees

**Fig. 4:**
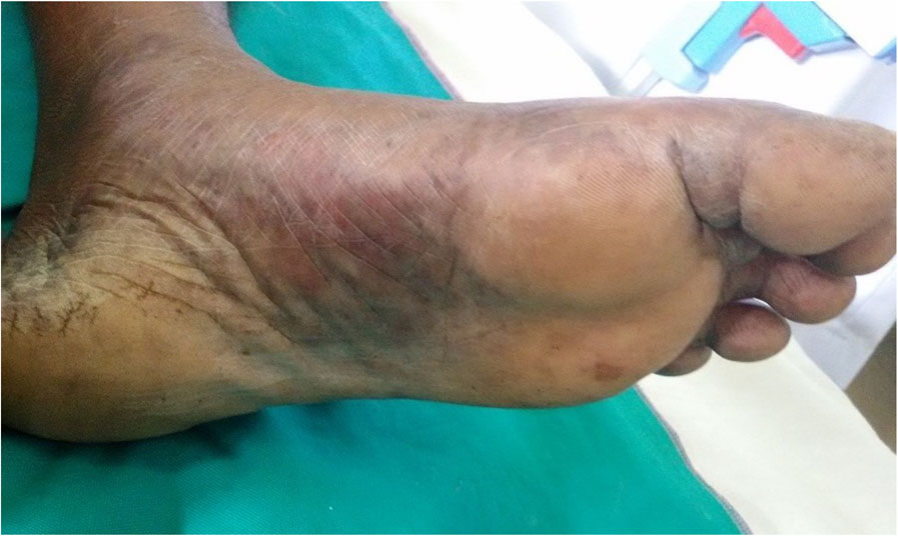
Evidence of subcutaneous vasculitis over the soles of the feet

However, despite the above treatment, she continued to deteriorate rapidly with worsening of symptoms. On the 2nd day of admission (10th day of illness) the patient rapidly developed a painful rash over the breasts (Fig. 1) which progressed to involve the rest of the body, mainly on the abdomen, upper limbs & hands (Fig. 2) and lower limbs (Fig. 3) with sparing of the face. Examination of the rash revealed multiple subcutaneous hemorrhagic skin lesions in a ‘fern leaf’ pattern together with a vasculitic rash over the soles (Fig. 4). She also had splinter hemorrhages, persistent tachycardia (120/min), tachypnea (33/min) and a high fever indicative of systemic inflammatory response syndrome.

At this stage, the blood picture showed fragmented red cells and thrombocytopenia suggesting early DIC. Her hemoglobin dropped to 8.6 g/dL from 10 g/dL and the platelet count was 76 × 10^3^/mL, APTT 40 s with INR 1.3 and elevated D dimers of 1.2 ng/ml (normal < 0.5 ng/ml). However, she did not have active bleeding from elsewhere. The clinical picture together with the rash led to the clinical suspicion of infective endocarditis, meningococcal or gonococcal septicaemia, severe staphylococcal or streptococcal sepsis, acute flare of connective tissue disease with vasculitis, cryoglobulinemia, a hemorrhagic form of leptospirosis, or rickettsial infection.

**Fig. 5:**
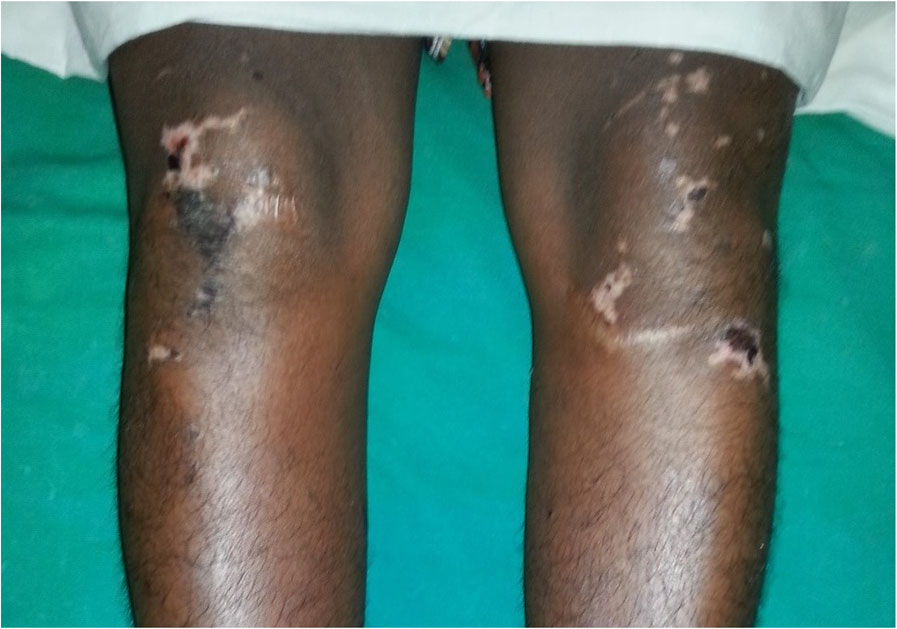
Peeling of the skin over the areas of subcutaneous necrosis

**Fig. 6:**
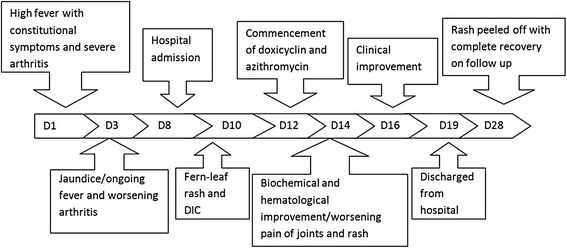
The timeline diagram of the evolution of illness

Transesophageal and transthoracic echocardiograms did not show evidence of infective endocarditis or myocarditis. ASOT titre was <200 Units. Chest radiograph was normal, and ultrasound scan of the abdomen revealed mild hepatomegaly. Intravenous vancomycin, oral doxycycline and azithromycin were added on the 3rd day of admission to cover severe gram positive sepsis and rickettsial infections. The IFA-IgG titre against *Rickettsia conorii* Ag was positive at a titre of 1: 8192 and leptospira antibodies were negative by both Microscopic Agglutination Test and ImmuneMed Leptospira Rapid Immuno-Chromatographic assay for qualitative detection of IgM, IgG antibodies to Leptospira. The patient showed gradual improvement with reduction in fever by the 2^nd^day of doxycycline (5th day of admission) and had a complete recovery from acute kidney and liver injury by the 4th treatment day. However, her skin rash and the joints became very painful over the 2nd-4th days of treatment. All these resolved completely within 5 days of treatment (8th day of admission), and she was discharged on the 11^th^day of admission. On review after one week of discharge, her rash had started to desiccate and peel off (Fig. 5). The timeline diagram of the evolution of illness is give in Fig. 6.

## Discussion

This otherwise healthy female who presented with an acute febrile illness complicated by severe small joint and large joint arthritis, jaundice, acute kidney injury and DIC later developed a subcutaneous hemorrhagic and a vasulitic skin rash. Her acute illness was later confirmed as SFGR and had a complete recovery with anti-rickettsial antibiotics.

At presentation, her clinical presentation mimicked palindromic rheumatoid arthritis or other connective tissue diseases with systemic involvement. However, exposure to floods suggested the possibility of acute leptospirosis. Neck stiffness and the haemorrhagic rash with rapid progressive illness suggested other agents of severe sepsis such as meningococci, gonococci, staphylococci or gram negative bacilli. However, the occurrence of the rash later in the illness and non-responsiveness to broad spectrum antibiotics suggested the possibility of rickettsial infection. Therefore, she was treated with anti-rickettsial antibiotics and had a rapid clinical response. She had a strong antibody response to SFGR demonstrated by IFA using *R. conorii* antigen, confirming the diagnosis. On specific questioning, she revealed that she had tick bites quite frequently when caring for the two dogs.

The clinical spectrum of severity of rickettsial infections ranges from subclinical to fatal and presents with fever and may progress to various organ involvement such as encephalitis, interstitial pneumonia/ARDS, acute renal failure or with multiple organ failure. Furthermore, we have encountered patients with rickettsial illness, either SFGR or ST with illness mimicking acute rheumatic disease [13]. Although most of the patients with SFG rickettsial disease have an unremarkable course, patients who develop complications related to organ involvement may suffer severe illness with mortality up to 2.5% among diagnosed cases [14]. Classical risk factors for high mortality or severe forms of infection include advanced age, chronic alcoholism, glucose-6-phosphate-dehydrogenase deficiency, prior prescription of an inappropriate antibiotic, particularly sulfonamides, or delay in the treatment [15]. Therefore, early clinical suspicion and empiric treatment with anti-rickettsial antibiotics are important in reducing duration of illness and mortality. It is important that rickettsial infections should always be considered in the differential diagnosis of acute febrile illness in endemic areas.

As there are no readily available rapid diagnostic tests for rickettsial illness, confirmation of the illness during its acute phase in most endemic settings is difficult. However, presence of clinical manifestations such as eschar or vasculitic rash favors the presumptive diagnosis and early institution of empirical anti-rickettsial antibiotics.

Typical cutaneous manifestations of SFGR include a discrete maculapapular rash which usually appears towards the end of the first week of illness and involves the trunk, limbs, palms and soles, however spares the face. The rash tends to be more prominent at the time the patient is having fever and fades in between fever spikes. Other rare forms of skin rash have been described, and involve a dusky erythematous hue, distributed mainly on the limbs, back of the chest, anterior abdomen and soles [2]. Severe rashes associated with SFG rickettsioses include digital gangrene and extensive skin necrosis described as PF [9–11]. While the digital gangrene may result in auto-amputation of toes [8], the skin involvement in PF results in blackish discoloration of skin that desiccates and peels off with complete recovery or hypertrophic scar formation after healing [9].

The occurrence of PF or ‘fern leaf’ type of rash seems to be rare in rickettsial infections. While PF has been described mainly in the children [9–11], ‘fern leaf rash has been observed in several elderly patients with SFGR in the central province of Sri Lanka [3, 6, 7, 12]. However, this young female patient very rapidly developed a widespread ‘fern-leaf’ pattern rash over the 10th and 11th days of illness together with DIC suggesting early PF. It was noted predominantly over the breasts and proximal limbs with facial sparing. The rash was painful and tender. The tenderness increased during the first three days after commencement of specific treatment and later improved in parallel with the other clinical manifestations. She also had splinter hemorrhages and a vasculitic rash on the palms and soles. This patient did not have an eschar or marks of tick bites and eschars are an uncommon feature in patients with SFGR compared to scrub typhus in Sri Lanka [16].

Today, the expansion of international and local travel, recreational activities, eco-tourism, animal transportation together with effects of global warming and migration of birds have resulted in vector borne diseases emerging as global threats. Non-availability of rapid diagnostic tests for most neglected tropical vector borne diseases has added to the clinical challenge in both the resource poor tropics as well as in the developed world. Until such time, as effective early diagnostic methods are available, the most important aspect in the management of such illnesses is the vigilance of clinicians. It is crucial to formulate the most likely differential diagnosis based on the clinical presentation of acute undifferentiated febrile illness and the epidemiological data. In order to achieve this goal, attention paid to the chronology of events such as progression of the symptoms or appearance of signs such as skin rash plays a vital role. Here we aim to highlight the unusual clinical presentation of this patient and the importance of the late onset skin rash that alerted the physicians to the possible diagnosis of SFGR.

## Conclusion

We feel that clinicians should be aware of the unusual clinical presentations such as purpura fulminans and ‘fern-leaf’ pattern necrotic skin rash of SFGR infection. Such knowledge would not only benefit those who practice in tropics with limited diagnostic facilities but also would improve the management of acute febrile illness in returning travelers who visit endemic areas.

## Abbreviations

ALT: Alanine Transaminase
APTT: Activated Partial Thromboplastin Time
ARDS: Adult Respiratory Distress Syndrome
ASOT: Anti-streptolysin O titre
AST: Aspartate transaminase
CRP: C-Reactive Protein
DIC: Disseminated Intravascular Coagulation
ESR: Erythrocyte Sedimentation Rate
IFA: Indirect Immunofluorescence antibody
IgG/IgM: Immunoglobin G/M
INR: International Normalization Ratio
PF: Purpura fulminans
SFGR: Spotted fever group rickettsioses
ST: Scrub typhus
